# TRAIL and proteasome inhibitors combination induces a robust apoptosis in human malignant pleural mesothelioma cells through Mcl-1 and Akt protein cleavages

**DOI:** 10.1186/1471-2407-13-140

**Published:** 2013-03-22

**Authors:** Bao-Zhu Yuan, Joshua Chapman, Min Ding, Junzhi Wang, Binghua Jiang, Yon Rojanasakul, Steven H Reynolds

**Affiliations:** 1 National Institutes for Food and Drug Control, Beijing, 100050, China; 2 National Institute for Occupational Safety and Health, CDC, Morgantown, WV, 26505, USA; 3 Mary Babb Randolph Cancer Center, West Virginia University, Morgantown, WV, 26506, USA; 4 Department of Cell Biology, National Institute for Food and Drug Control, 2 Tiantan Xili, Dongcheng District, Beijing, 100038, China

**Keywords:** Malignant pleural mesothelioma, Apoptosis, Trail, Proteasome inhibitor, Mcl-1, Akt

## Abstract

**Background:**

Malignant pleural mesothelioma (MPM) is an aggressive malignancy closely associated with asbestos exposure and extremely resistant to current treatments. It exhibits a steady increase in incidence, thus necessitating an urgent development of effective new treatments.

**Methods:**

Proteasome inhibitors (PIs) and TNFα-Related Apoptosis Inducing Ligand (TRAIL), have emerged as promising new anti-MPM agents. To develop effective new treatments, the proapoptotic effects of PIs, MG132 or Bortezomib, and TRAIL were investigated in MPM cell lines NCI-H2052, NCI-H2452 and NCI-H28, which represent three major histological types of human MPM.

**Results:**

Treatment with 0.5-1 μM MG132 alone or 30 ng/mL Bortezomib alone induced a limited apoptosis in MPM cells associated with the elevated Mcl-1 protein level and hyperactive PI3K/Akt signaling. However, whereas 10–20 ng/ml TRAIL alone induced a limited apoptosis as well, TRAIL and PI combination triggered a robust apoptosis in all three MPM cell lines. The robust proapoptotic activity was found to be the consequence of a positive feedback mechanism-governed amplification of caspase activation and cleavage of both Mcl-1 and Akt proteins, and exhibited a relative selectivity in MPM cells than in non-tumorigenic Met-5A mesothelial cells.

**Conclusion:**

The combinatorial treatment using TRAIL and PI may represent an effective new treatment for MPMs.

## Background

Malignant pleural mesothelioma (MPM) is an aggressive malignancy arising from the mesothelium-lined surfaces of the pleural cavities following exposure to asbestos [1]. Approximately 3,000 new MPM cases are diagnosed each year in the United States, with an even higher number worldwide. Although measures have been put in place to limit further asbestos exposure, the long latency of disease development post exposure has resulted in a dramatically increasing current incidence of MPM [2].

MPM is extremely resistant to most chemotherapy regimens examined and not responsive primarily to radiation therapy in general [3,4]. Pemetrexed (Alimta) and cisplatin combination was shown to be the best chemotherapy regimen for MPM examined so far. However, median survival with this therapy was less than one year and the response rate was lower than fifty percent [5]. Targeted therapies, such as anti-angiogenic drugs and inhibitors of the epidermal growth factor receptor (EGFR) tyrosine kinase, have proved to be equally ineffective in prolonging MPM patient survival despite substantial over-expression of the relevant molecular targets in MPM cells [6]. Recent clinical studies showed that tri-modality therapy with radical pleurectomy, chemotherapy with cisplatin plus pemetrexed or cisplatin plus gemcitabine, and radiation can significantly increase median survival rate of MPM patients up to 30 months from previously less than 12 months with mono-therapy [7,8]. Nevertheless, it is still urgent to search for effective new treatments for MPM.

Evasion of apoptosis induction is believed to be a common scheme employed by tumor cells to resist various treatments [9]. Vast majority of apoptosis are executed by caspases, which are either the initiator caspases or effector caspases depending on timing of activation [10]. The intrinsic or mitochondrial apoptosis pathway plays a fundamental role in various types of apoptosis. Upon activation, the mitochondria release Cytochrome c (Cyto c) and Smac/Diablo (Smac) into the cytosol, leading to sequential activation of caspases 9 and 3/7 and ultimately apoptotic cell death [11,12]. The intrinsic pathway is regulated mainly by the antiapoptotic members of the Bcl-2 family proteins, such as Bcl-2, Bcl-XL and Mcl-1 [13]. The extrinsic pathway is responsible for death receptor-mediated apoptosis. Upon binding by the TNFα family proteins, death receptor(s) recruit/activate caspase 8/10 through FADD leading to activation of caspase 3/7.

The PI3K/Akt signaling provides important survival mechanisms in tumor cells by promoting growth and metastasis, and reducing sensitivity to chemotherapies [14,15]. The hyperactive PI3K/Akt signaling is commonly seen in MPMs likely due to frequent loss of PTEN expression [16]. It is also closely associated with asbestos exposure and SV40 virus infection in the pathogenesis of MPM [17-19]. PI3K/Akt has thus been proposed to be a therapeutic target for MPM [20].

Proteasome inhibitors (PIs) are becoming potential therapeutic agents for various types of human cancer that are refractory to conventional chemotherapies [21]. The therapeutic effects of PIs are attributable to their ability to induce apoptosis [22]. Previous findings have demonstrated that PIs can induce apoptosis through activating the intrinsic apoptosis pathway, which is under the regulation by the elevated Mcl-1 protein level following proteasome inhibition [23,24]. However,PIs can also overcome Mcl-1’s regulation through activation of a highly efficient positive feedback mechanism (PFM),which amplifies caspase activation subsequently causing Mcl-1 protein cleavage [23]. The effect of the PFM is achieved through linking the initially activated intrinsic pathway to the extrinsic pathway, thus forming an apoptosis signaling loop and ensuring quick complete apoptotic cell death [23]. PIs can only induce a limited apoptosis in MPM cells most likely due to their inability to activate PFM and to cleave Mcl-1 protein [24].

The TNF-related apoptosis inducing ligand (TRAIL) is a 281-amino acid proapoptotic cytokine of the TNFα family. After binding to death receptor DR4 or DR5, the TRAIL protein activates the extrinsic pathway through recruiting/activating caspases 8/10 [25,26]. TRAIL plays an important role in tumor immune surveillance system by selectively inducing tumor cell apoptosis while leaving normal cells unharmed [25,26]. However, tumor cells may develop resistance mechanisms to TRAIL-induced apoptosis at different points along the TRAIL signaling pathway [25,26].

The combinatorial treatment with TRAIL and PIs can significantly increase the induction of apoptotic cell death in some human cancers, including multiple myeloma, renal carcinoma and NSCLC cells, which were not sensitive to either TRAIL alone or PI alone treatment [23,27]. However, no attempt has been made to address the effect of TRAIL and PI combination in MPM cells. In this study, we show that MPM cells are generally resistant to either PI or TRAIL alone treatment. Both the hyperactive PI3K/Akt signaling and the concurrently elevated Mcl-1 are responsible for the resistance to PI. However, the TRAIL and PI combination can induce a robust apoptotic cell death in all MPM cells. Moreover, it is believed that the significantly enhanced activity is achieved through activating the PFM [23] and subsequently cleaving proteins Mcl-1 and Akt. Most importantly, such effect exhibits a relative selectivity in MPM cells than in non-tumorigenic mesothelial cells, suggesting that TRAIL and PI combination may represent an effective new treatment for MPMs.

## Methods

### Materials

*Cell lines:* Human MPM cell lines NCI-H2052, -H28 and -H2452, the sarcomatoid, epithelial and biphasic (mixed) types of MPM, respectively, and non-tumorigenic Met-5A mesothelial cell line were purchased from ATCC and cultured in RPMI 1640 medium supplemented with 10% FBS. *Chemicals:* proteasome inhibitor MG132, caspase inhibitors for wide spectrum caspases (Z-VAD-fmk), caspase 3 (Z-DQMD-fmk), caspase 8 (Z-IETD-fmk), caspase 9 (Z-LEHD-fmk) and caspase 10 (Z-AEVD-fmk), and a negative control (Z-FA-fmk), PI3K specific inhibitor LY294002, were from EMD-CalBiochem (San Diego, CA); proteasome inhibitor Bortezomid was from ChemieTek (Indianapolis, IN); Mcl-1 siRNA and a negative control siRNA were from Santa Cruz (Santa Cruz, CA); Soluble recombinant human TRAIL protein was from R&D Systems (Minneapolis, MN). *Antibodies*: the antibodies against caspases 3, 7, 9 and 10, PARP, Akt, phospho-Akt at Ser473 (or P-Akt), STAT3, phospho-STAT3 at Tyr705 (or P-STAT3) were from Cell Signaling (Danvers, MA); the antibodies against caspase 8, Mcl-1 and Bcl-XL were from Santa Cruz; the antibodies against Bcl-2 and actin were from Sigma (Milwaukee, WI).

### Western blotting

Procedures of conventional Western blotting were followed to monitor expression and/or cleavage of apoptosis-related proteins in MPM cells after various treatments. RIPA buffer supplemented with proteinase inhibitor cocktail (Sigma, Milwaukee, WI) was used to collect cell lysates and 10-14% PAGE gels were used to separate samples before transferring them onto nitrocellulose membrane. ECL Advance Western Blotting Detection Kit (GE Healthcare, Piscataway, NJ) was used for detecting signals.

### Cell viability assay

A previously described procedure using WST-1 reagent (Roche, Indianapolis, IN) was followed to measure cell viability [24]. Briefly, after various treatments, 0.5-1 × 10^4^ cells growing in each well of a 96-well microplate were incubated with 10 μl of WST-1 reagent (Roche,Indiannapolis, IN) for 1 to 4 hours. Triplicate wells were set up for each sample in each experiment. The increase of absorbance at 420 to 480 nm relative to the blank control was measured for each sample using a microplate spectrophotometer.

### Flow cytometry assay

Sub-G0/G1 fraction reflecting DNA fragmentation was detected in a flow cytometry assay as described previously [24,28]. Briefly, approximately 1 × 10^5^ cells were collected after treatment, fixed in 70% ethanol, and stained with propidium iodide, and DNA content was determined on a flow cytometer (FACSCalibur; BD Biosciences, San Jose, CA).

### Akt gene construct and transfection

Mouse wild type Akt (wtAkt) or constitutively active Akt (myristylated Akt, myr-Akt) cDNA [29] was constructed in pcDNA3.1Zeo(+) vector and stably transfected into NCI-H2452 cells following the previously described procedures [30,31]. The cells selected by Zeocin (25–100 μg/ml) were tested for their responses to different apoptosis stimuli.

### Mcl-1 silencing

The procedures of siRNA transfection described previously were followed to transfect Mcl-1 siRNA or control siRNA into NCI-H28 cells [24]. At 36 h after siRNA transfection, tumor cells were treated and then analyzed for their responses to different apoptosis inductions. The siRNA silencing experiment was repeated at least twice.

### Semi-quantitative reverse transcription-PCR (RT-PCR)

Polyadenylated RNA was extracted from NCI-H28 cells using Trizol reagent and magnetic oligo (dT) beads, and then used in RT-PCR for detecting Akt gene transcription. GAPDH mRNA expression was used as a control in semi-quantitation of PCR products. Primer sequences for detecting Akt are 5^′^-gctacttcctcctcaagaatgatggc-3^′^ and 5^′^-gcagcttcaggtactcaaactcgttc-3^′^ and for GAPDH are 5^′^-ggctctccagaacatcatccctgc-3^′^ and 5^′^-gggtgcgctgttgaagtcagagg-3^′^.

### Statistics

Data for cell viabilities were expressed as the means ± SD of at least two separate experiments. Comparison between group means was assessed using a one-way ANOVA with the Newman-Keuls posttest (GraphPad Prism 3.0 Software, Inc., San Diego, CA). *P* < 0.05 was considered significant.

## Results

### Proteasome inhibitor MG132 alone or TRAIL alone induces a limited apoptosis in MPM cells

**Figure 1:**
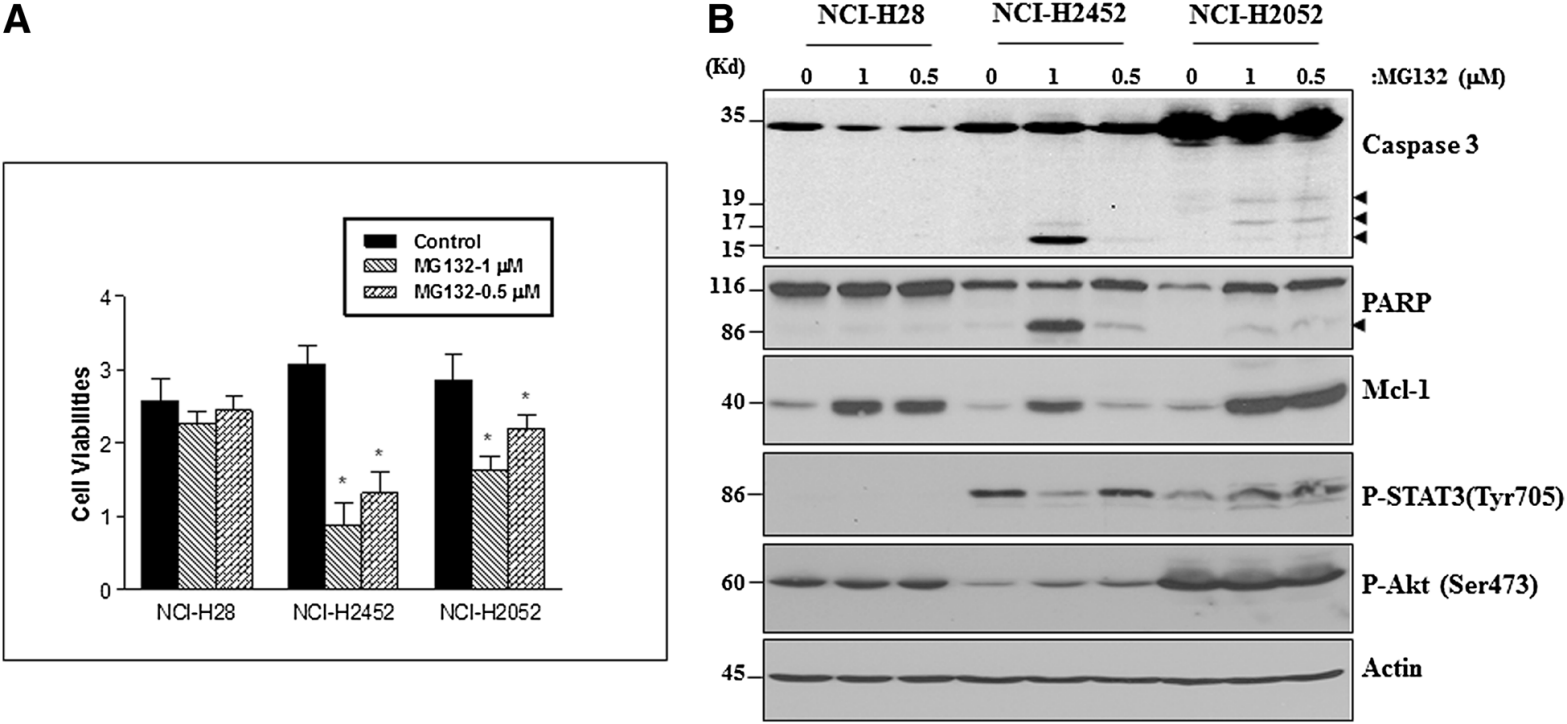
**MG132 induces a limited apoptosis in MPM cells. (A)** Cell viability assay using WST-1 reagent indicates that 0.5-1 μM MG132 induces a significant cell death at 72 h after treatment in both NCI-H2452 and NCI-H2052 cells, but not in NCI-H28 cells. Values are expressed as the means ± SD of two experiments. *p < .05 *versus* control. **(B)** Western blotting demonstrates that 0.5-1 μM MG132 induces protein cleavages for caspase 3 and PARP at 42 h after treatment in NCI-H2452 and NCI-H2052 cells, but not in NCI-H28 cells. The treatments cause a significant Mcl-1 protein elevation in all three MPM cell lines. NCI-H28 and NCI-H2052 cells exhibit a much higher P-Akt protein level than NCI-H2452 cells, whereas the P-STAT3 protein is only detectable in NCI-H2452 and NCI-H2052 cells. Protein cleavage fragments in Western blots are indicated by arrows.

In this study, we observed that 0.5-1 μM MG132 induced only a limited cell death and protein cleavages in both NCI-H2452 and NCI-H2052 cells with a greater effect seen in NCI-H2452 cells, but no effects in NCI-H28 cells (Figure 1A & B). Treatment with MG132 significantly elevated Mcl-1 protein level in all three MPM cell lines with a more significant elevation seen in NCI-H28 and NCI-H2052 cells (Figure 1B), supporting that Mcl-1 is a major regulatory protein against the PI-induced apoptosis in MPM cells [24].

Since the activated Akt, phospho-Akt (P-Akt), or STAT3, phospho-STAT3 (P-STAT), confers resistance to apoptosis through up-regulating the antiapoptotic proteins, such as Mcl-1 in cancer cells [32], to further understand the resistance of MPM cells to PI-induced apoptosis, we examined P-Akt and P-STAT3 levels in the PI-treated MPM cells. A high level of P-Akt was observed in NCI-H28 and NCI-H2052 cells, but not in NCI-H2452 cells, while a high level of P-STAT3 was detected in NCI-H2452 and NCI-H2052, but not in NCI-H28 cells, suggesting that P-Akt is more likely involved in regulating the PI-induced apoptosis than P-STAT3 in MPM cells (Figure 1B).

**Figure 2:**
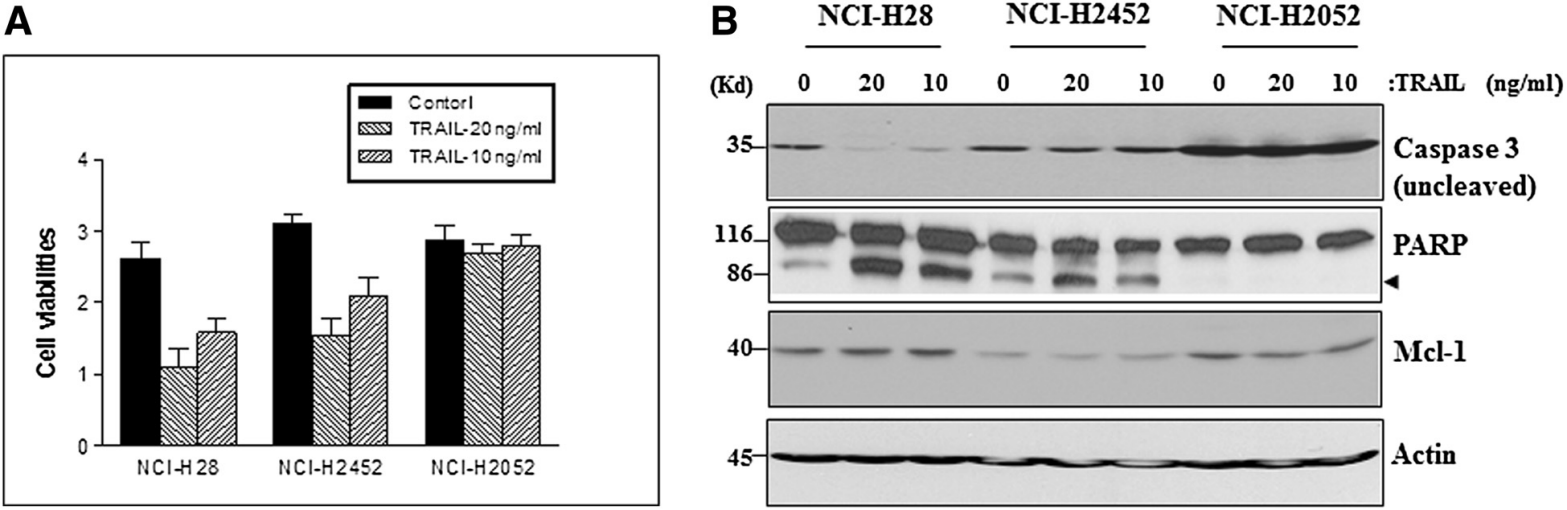
**TRAIL induces a limited apoptosis in MPM cells. (A)** Cell viability assay using WST-1 reagent indicates that 10–20 ng/ml RAIL induces a significant cell death at 72 h after treatment in NCI-H28 and NCI-H2452 cells, but not in NCI-H2052 cells. Values are expressed as the means ± SD of two experiments. *p < .05 *versus* control. **(B)** Western blotting demonstrates that 10–20 ng/ml TRAIL induces protein cleavage for caspase 3 and PARP at 42 h after treatment in NCI-H28 and NCI-H2452 cells, but not in NCI-H2052 cells. Protein cleavage fragments in Western blots are indicated by arrows.

In addition, we found in this study that 10–20 ng/ml TRAIL alone treatment induced a mild cell death and protein cleavages in both NCI-H28 and NCI-H2452 cells, but not in NCI-H2052 cells (Figure 2A & B). TRAIL alone treatment however showed no effect on Mcl-1 protein expression (Figure 2B).

### The TRAIL and PI combination induces a robust apoptosis in MPM cells through the PFM-governed caspase activation

**Figure 3:**
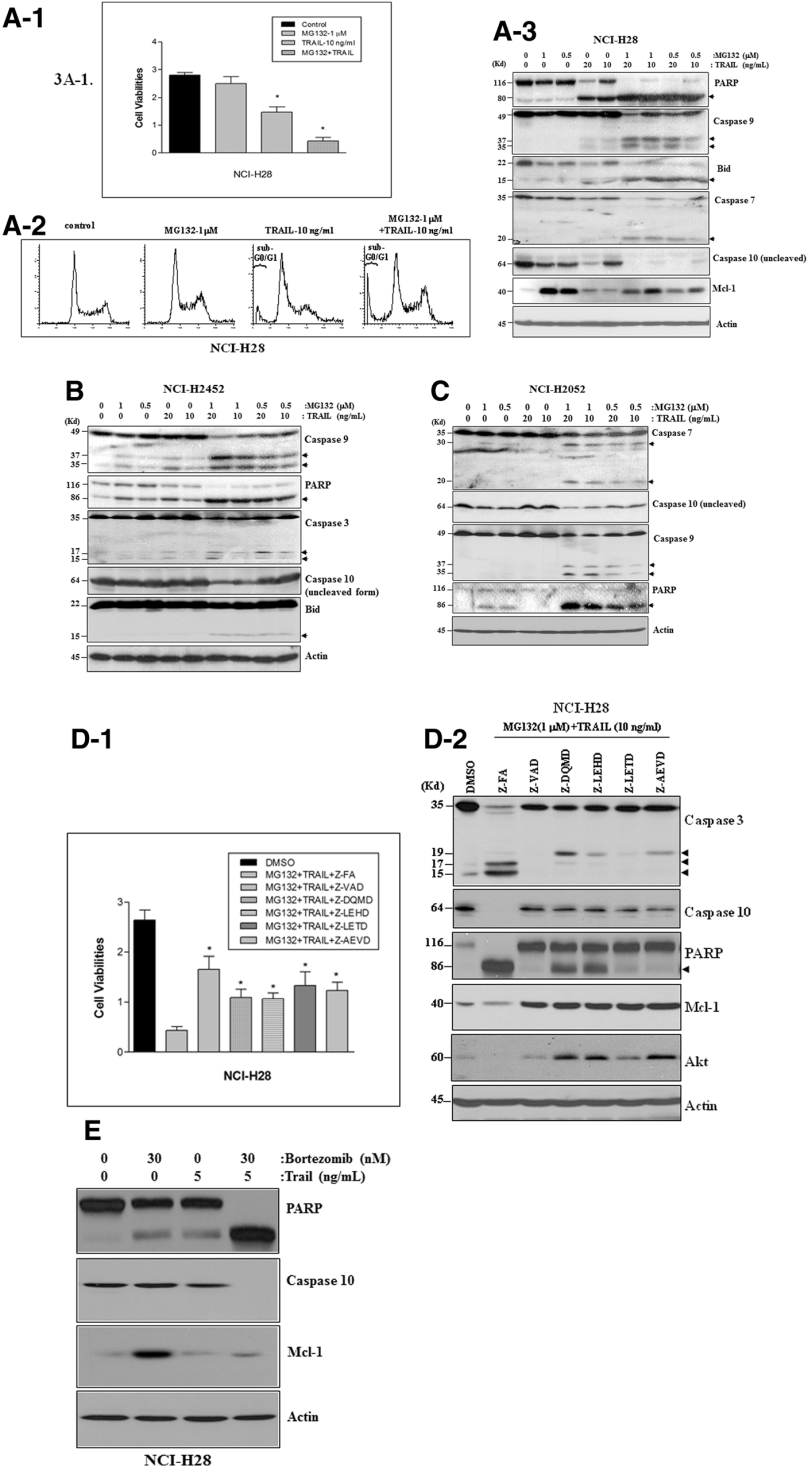
**TRAIL and MG132 (or Bortezomib) combination induces a robust apoptosis in MPM cells. (A)** The combinatorial treatment induces a robust apoptosis in NCI-H28 cells. **A-1**: Cell viability assay using WST-1 reagent indicates that 10 ng/ml TRAIL and 1 μM MG132 combination induces a dramatic cell death at 72 h after treatment. Values are expressed as the means ± SD of two experiments. *p < .05 *versus* control; **A-2**: Flowcytometry assay demonstrates that the two-agent combination induces a significant increase in DNA damage as indicated by the sub G0/G1 peak; **A-3**: Western blotting reveals that the two-agent combination induces a significantly increased protein cleavage for caspases 7, 9 and 10, Bid and PARP in NCI-H28 cells at 24 h after treatment. **(B & C)** Western blotting demonstrates that the two-agent combination significantly enhanced apoptosis in both NCI-H2452 and NCI-H2052 cells, as indicated by the enhanced protein cleavage for caspases 3, 9 and 10, Bid and PARP in NCI-H2452 cells at 36 h after treatment (**B**) and caspases 7, 9 and 10 and PARP in NCI-H2052 cells at 40 h after treatment (**C**). **(D)** The two-agent-induced-apoptosis is caspase-dependent. Specific inhibitor for the broad spectrum caspases (Z-VAD), caspase 3 (Z-DQMD), caspase 9 (Z-LEHD), caspase 8 (Z-IETD) or caspase 10 (Z-AEVD) reduces the two-agent-induced cell death (3**D-1**), as revealed by cell viability assay at 72 h after treatment, and protein cleavage for caspases 3 and 10, PARP, Mcl-1 and Akt, as demonstrated by Western blotting at 36 h after treatment. Z-FA is used as a negative control for all caspase inhibitors. *p < .05 *versus* control. Protein cleavage fragments in Western blots are indicated by arrows. (**E**) TRAIL and Bortezomib combination induces a robust apoptosis in NCI-H28 cells with a significant cleavage for both caspase 10 and Mcl-1 proteins.

Following single agent alone treatment, we observed that the combinatorial treatment with 0.5-1 μM MG132 and 10–20 ng/ml TRAIL resulted in a dramatically increased cell death and protein cleavages in all three MPM cell lines with a greater significance seen in NCI-H28 cells (Figure 3A, B & C). Among the proteins undergoing cleavage are PARP, Bid and caspases 3, 7, 9,10 and Mcl-1 proteins, indicating that both the intrinsic and extrinsic apoptosis pathways were activated by the combinatorial treatment.

To further determine the caspase-dependent nature of the combinatorial treatment, we chose NCI-H28 cells for co-treatment using 1 μM MG132 plus 10 ng/ml TRAIL and 10 μM individual specific inhibitor for caspase 3, 9, 8 or 10. It was found that inhibition of each individual caspase significantly reduced both cell death and protein cleavages (Figure 3D), demonstrating that the proapoptotic activity of the combinatorial treatment was caspase dependent with involvement both intrinsic and extrinsic apoptosis pathways.

It was further observed that, while TRAIL alone induced a limited caspase 10 cleavage only in NCI-H28 cells and showed no effect on Mcl-1 protein level, the combinatorial treatment dramatically enhanced protein cleavage for both caspase 10 and Mcl-1, especially in NCI-H28 cells (Figure 3A). In addition, the increased protein cleavage for both caspase 10 and Mcl-1 was completely blocked by the specific inhibitor of each individual caspase (Figure 3D), supporting that the robust apoptosis was the consequence of activation of the PFM, which linked both the intrinsic and extrinsic pathways together leading to quick completion of apoptosis [23].

To test whether other PIs could exhibit a similar robust proapoptotic activity when used in combination with TRAIL, we chose NCI-H28 as a representative in the treatment with 30 nM Bortezomib and 5 ng/ml TRAIL. It was found that the new combinatorial treatment also induced a robust apoptosis in MPM cells with significant protein cleavage for both Mcl-1 and caspase 10, whereas each single agent induced only a minimal apoptosis (Figure 3E), suggesting that the induction of robust apoptosis is a general feature of the PI and TRAIL combination treatment in MPM cells.

### Reduction of the P-Akt level contributes to the proapoptotic activity of the combinatorial treatment

**Figure 4:**
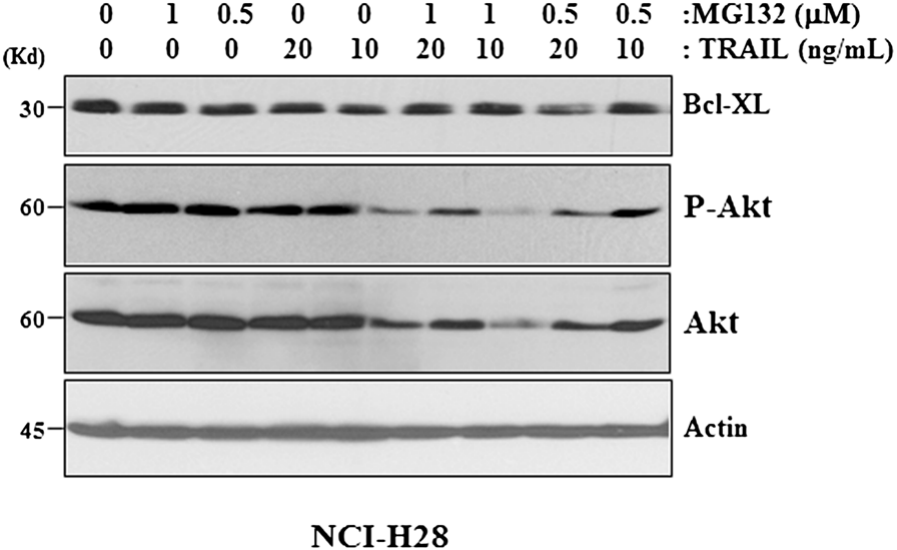
**Proapoptotic activities of TRIAL and MG133 combination treatment.** Treatment with 10–20 ng/ml TRAIL and 0.5-1 μM MG132 combination reduces protein level for both phospho- and non-phospho-Akt in NCI-H28 cells, as revealed by Western blotting at 24 h after treatment.

**Figure 5:**
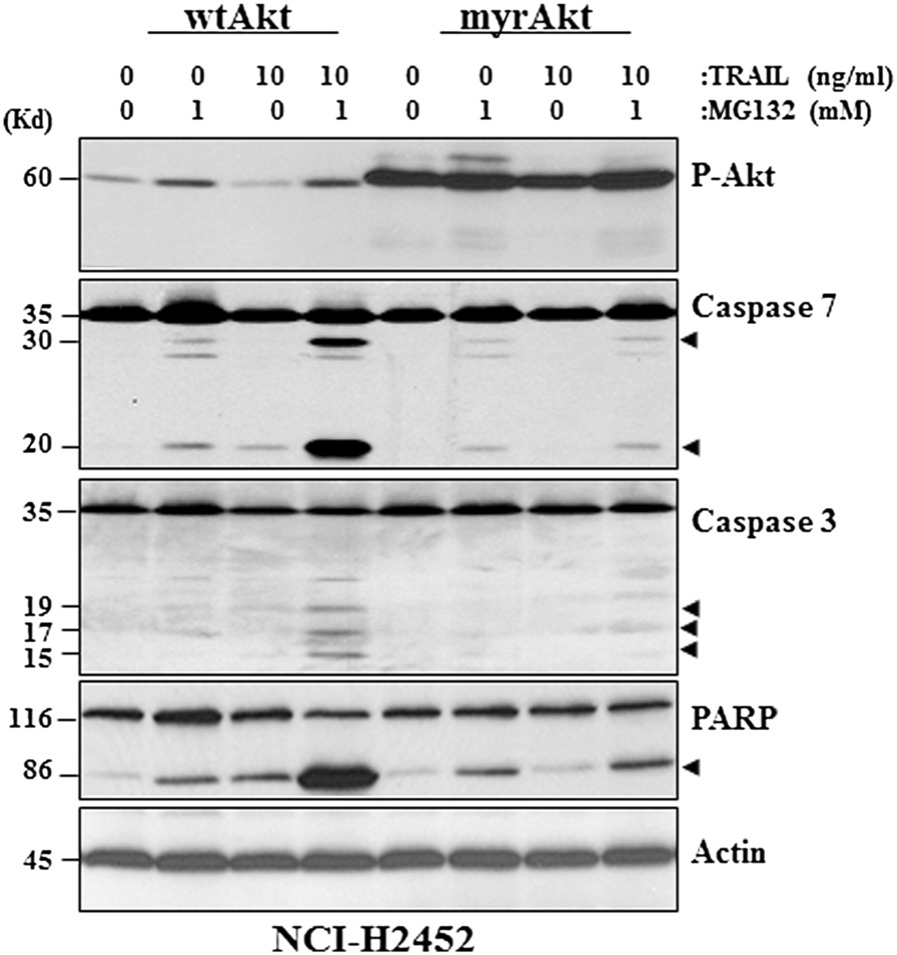
**The sustained Akt activity confers resistance to MG132, TRAIL or MG132 plus TRAIL-induced apoptosis to NCI-H2452 cells.** Western blotting demonstrates that transfection of the constitutively active Akt (myrAkt) results in a sustained phosphorylation of Akt at Ser 473 residue and reduces protein cleavages for caspases 3 and 7 and PARP induced by 1 μM MG132, 10 ng/ml TRAIL or MG132 plus TRAIL.

In addition to the reduction of Mcl-1 protein level, the combinatorial treatment also significantly reduced P-Akt protein level in all three MPM cell lines (Figure 4, using NCI-H28 as a representative). To determine the involvement of P-Akt in the regulation of apoptosis induction revealed in this study, we transfected the constitutively active Akt (or myr-Akt) gene [29] into NCI-H2452 cells, which exhibit much lower endogenous P-Akt level and are relatively sensitive to either MG132 or TRAIL treatment (Figures 1 &2). It was found that the sustained Akt activity in myr-Akt-transfected cells significantly desensitized NCI-H2452 cells to the apoptosis induced by MG132, TRAIL or MG132 plus TRAIL (Figure 5), supporting that the active PI3K/Akt signaling serves as a critical resistance mechanism against either PI- or TRAIL-induced apoptosis in MPM cells and can be reduced by the combinatorial treatment.

### The Akt protein is also subjected to the caspase-dependent protein cleavage

**Figure 6:**
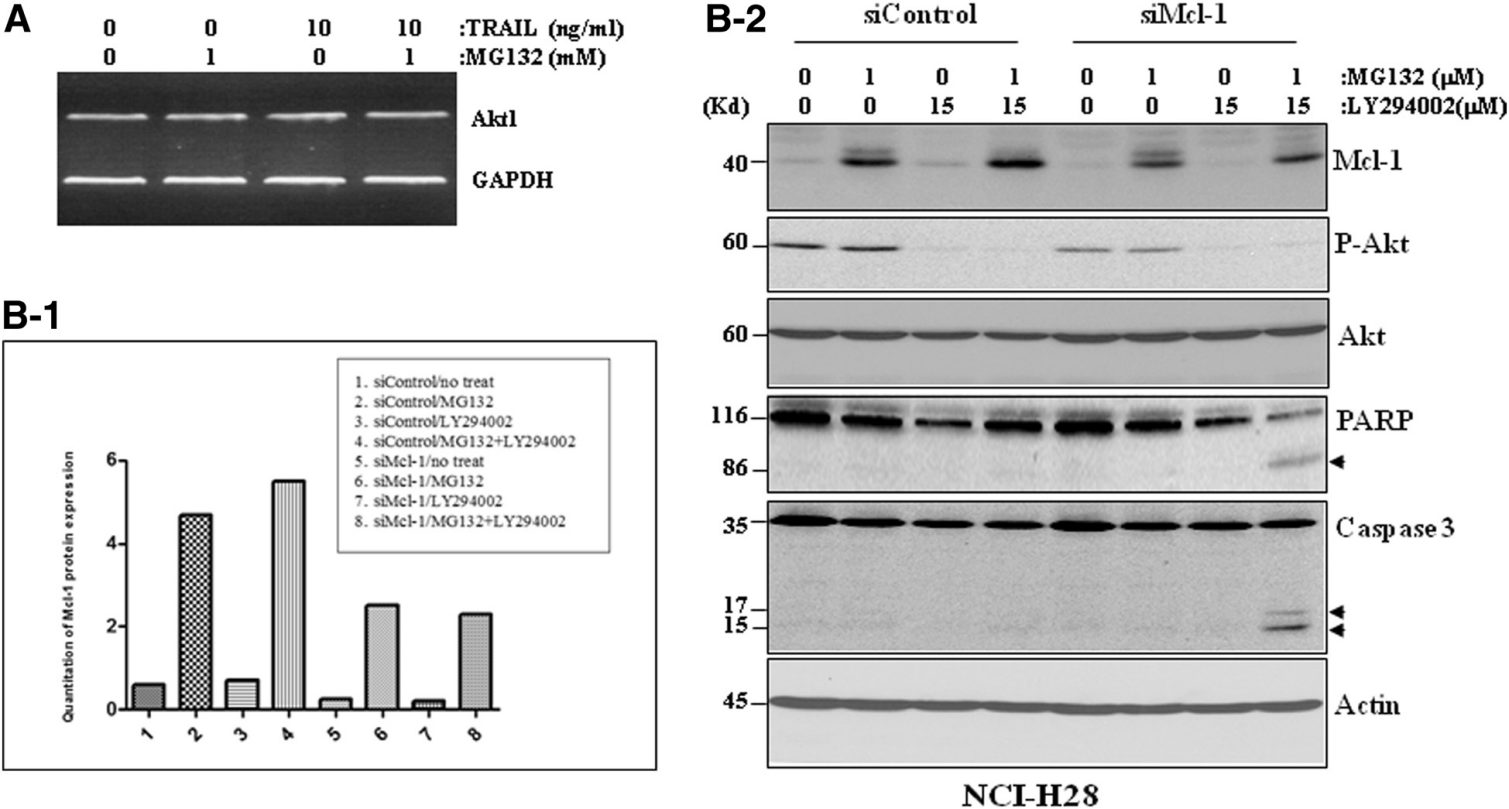
**The hyperactive PI3K/Akt signaling is responsible for the resistance of MPM cells to MG132-induced apoptosis.** (**A**) TRAIL and MG132 combination does not affect mRNA expression of the Akt gene in NCI-H28 cells, as indicated by a semi-quantitative RT-PCR at 36 h after treatment with 1 μM MG132, 10 ng/ml TRAIL or MG132 plus TRAIL. Expression of GAPDH is used as an internal control. (**B**) Simultaneous reduction of both P-Akt and Mcl-1 sensitizes NCI-H28 cells to MG132-induced apoptosis: Western blotting demonstrates that simultaneous reduction of P-Akt by 15 μM LY294002 and Mcl-1 by Mcl-1 siRNA, which results in approximately 50% reduction of Mcl-1 protein expression as determined by quantitative analysis using the online software Image J (**B-1**), can induce protein cleavages for caspase 3 and PARP in 1 μM MG132-treated NCI-H28 cells, whereas reduction of either P-Akt or Mcl-1 is not effective. Protein cleavage fragments in Western blots are indicated by arrows (**B-2**).

In the afore-mentioned experiment, it was observed unexpectedly that the protein level of both phospho- and non-phospho Akt were significantly reduced in NCI-H28 cells (Figure 4). Since a semi-quantitative RT-PCR experiment revealed that treatment with MG132, TRAIL or MG132 plus TRAIL did not affect Akt transcription, the possibility of down-regulation of gene transcription in reducing P-Akt level was thus excluded (Figure 6A). However, the reduction of P-Akt level was completely blocked by the specific inhibitors of caspases of either the intrinsic or extrinsic pathways (Figure 3D), thus demonstrating that, similar to Mcl-1, the Akt protein is also subjected to the PFM-governed caspase-dependent cleavage in MPM cells following the combinatorial treatment.

### PI3K/Akt and Mcl-1 represent two independent antiapoptotic mechanisms against the PI-induced apoptosis in MPM cells

LY294002, a PI3K specific inhibitor, inhibits activities of the PI3K/Akt signaling [33]. To determine the relationship between Akt and Mcl-1 in regulating PI-induced apoptosis, we treated NCI-H28 cells with 15 μM LY294002 and 1 μM MG132 following Mcl-1 siRNA transfection, which caused more than 50% reduction of Mcl-1 protein expression as determined by the online software Image J (Figure 6B-1). It was found that, although LY294002 completely blocked Akt phosphorylation, it failed to sensitize the cells to MG132 treatment, and Mcl-1 silencing alone did not sensitize the cells to MG132 as well. However, Mcl-1 silencing and LY294002 treatment together significantly increased sensitivity of NCI-H28 cells to the MG132-induced apoptosis (Figure 6B-2), suggesting that Mcl-1 and PI3/Akt represent two independent critical resistance mechanisms against PI-induced apoptosis.

### The TRAIL and PI combination exhibits a selective activity in MPM cells

**Figure 7:**
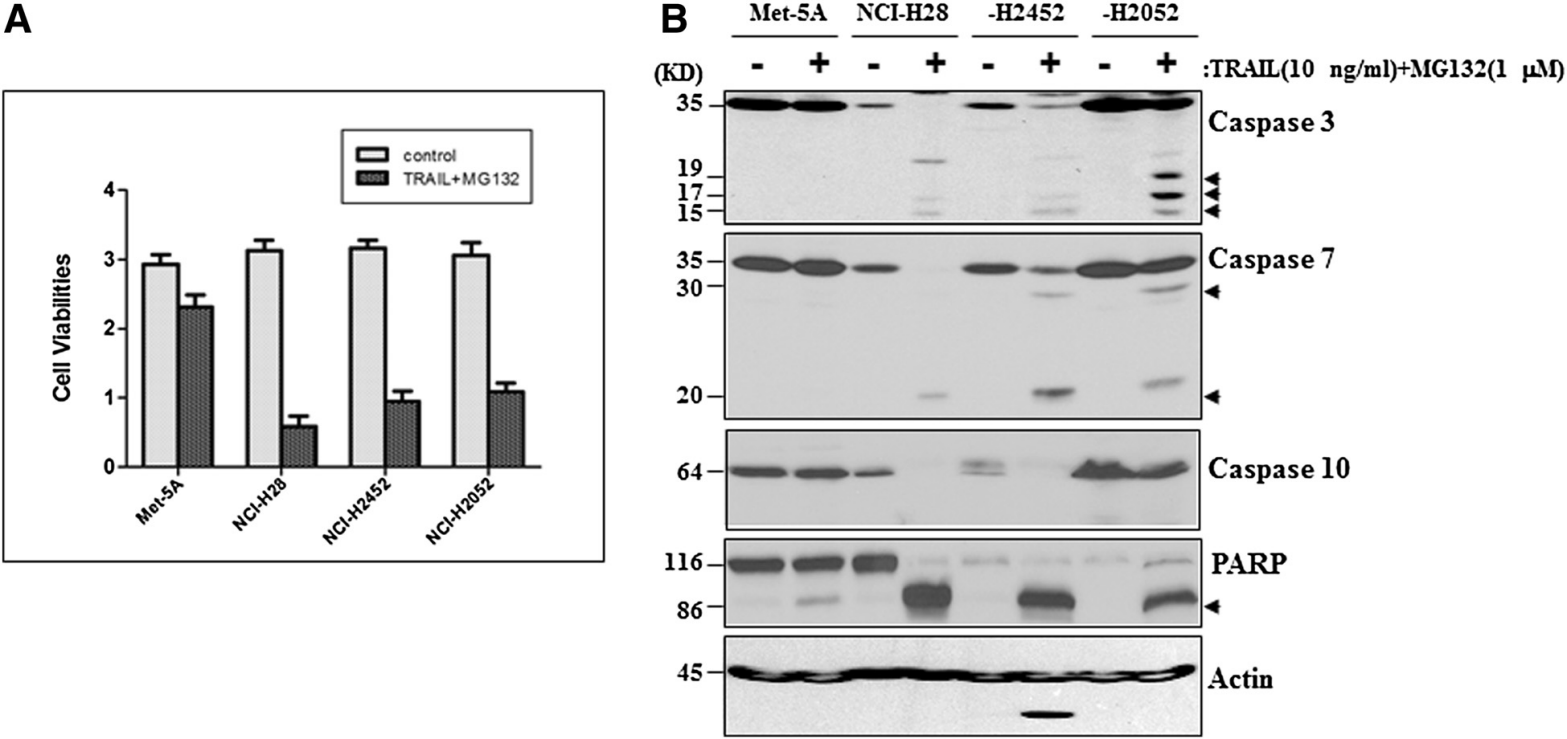
**TRAIL and MG132 combination exhibits a selective cell death in MPM cells.** (**A**) Cell viability assay using WST-1 reagent indicates that 10 ng/ml TRAIL and 1 μM MG132 combination induces a dramatic cell death at 72 h after treatment in all three MPM cells than in Met-5A cells. (**B**) Western blotting demonstrates that the combination of 10 ng/ml TRAIL with 1 μM MG132 induces a significant protein cleavage for caspases 3, 7 and 10 and PARP in all three MPM cell lines, but only induces a slightly visible cleavage for PARP in normal mesothelial Met-5A cell line. Protein cleavage fragments in Western blots are indicated by arrows.

In this study, we also compared apoptotic responses of non-tumorigenic mesothelial Met-5A cells with that of MPM cells to the combinatorial treatment. It was found that Met-5A cells exhibited a much lower amount of cell death (Figure 7A) and very limited protein cleavages (Figure 7B) than all three MPM cells to the combinatorial treatment, suggesting that the pro-apoptotic activity of the combinatorial treatment is more selective in MPM cells than in normal mesothelial cells.

## Discussion

MPM is a very aggressive malignancy with an extreme resistance to current treatments. Evasion of apoptosis induction due to the inactivated apoptosis machinery and/or up-regulated anti-apoptotic mechanisms, is largely responsible for its resistance to treatments [24]. A new treatment with ability to selectively activate the apoptotic machinery while inhibiting multiple antiapoptotic mechanisms in MPM cells will be a promising candidate treatment for MPM. This study reveals that the TRAIL and PI combinatorial treatment can induce a robust proapoptotic activity in MPM cells of all three major pathological types, but not in normal mesothelial cells. The robust activity is achieved through the PFM-governed caspase activation and subsequent protein cleavage of both Akt and Mcl-1, the two important anti-apoptotic proteins in MPM cells, therefore suggesting that the TRAIL and PI combination may serve as a promising new treatment for MPMs.

The PFM was characterized in a previous study as a highly efficient apoptosis induction mechanism, [23,24]. It conveys apoptosis signaling from the initially activated intrinsic pathway to the extrinsic pathway leading to cleavage/activation of caspase 10/8. The activated extrinsic pathway then forms an apoptosis signaling loop with the intrinsic pathway through the Bid protein and the mitochondria. Continuous signaling flow along this loop results in the amplified caspase activation and subsequent cleavage of the anti-apotpotic protein Mcl-1, thus ensuring a quick complete apoptotic cell death [23]. The protein cleavage for both caspase 10 and Mcl-1 can thus serve as a surrogate indicator of the PFM activation. Given the observation of a significant elevation in Mcl-1 protein level without caspase 10 cleavage in all three MPM cells following PI alone treatment, we conclude that PI alone treatment is insufficient to activate the PFM and can induce only a limited apoptosis in MPM cells.

The TRAIL protein induces apoptosis through binding death receptor DR4/DR5 and then recruiting/activating caspase 8/10. The TRAIL protein or death receptor activating antibodies have demonstrated a strong proapoptotic activity in various human cancer cells [25,26]. However, similar to PI alone, TRAIL alone can induce only a limited apoptosis in MPM cells probably due to its insufficiency to activate the PFM.

Although TRAIL and PI share a limited apoptotic activity in MPM cells, the sensitivity profiles of the MPM cells are different to these two agents: NCI-H28 cells are resistant to PIs but mildly sensitive to TRAIL, whereas NCI-H2052 cells are resistant to TRAIL but slightly sensitive to PI. Such difference reflects a mechanistic discrepancy in apoptosis induction between the two agents and encourages their combination for achieving an enhanced cell death. Indeed, the compensation can be seen during the activation of the PFM. Following the PFM model, it is believed that TRAIL can help PI induce sufficient activation of caspase 3/7 for initiating the PFM, resulting in caspase 10 activation while reducing Mcl-1 protein level through protein cleavage [23].

The Mcl-1 protein, among the antiapoptotic Bcl-2 and IAP family proteins, has been characterized to be a major protein regulating the PI-induced apoptosis in MPM cells [24]. This specificity is determined largely by the fact that Mcl-1 is a degradation target of the proteasome [34,35]. The Mcl-1 protein can also be cleaved by the activated caspase 3 [36]. It is thus not surprising to observe that proteasome inhibition in the absence of sufficient caspase 3 activation, as seen in PI alone treatment, elevates Mcl-1 protein level. However, the proteasome inhibition with sufficient caspase 3 activation, as seen in the combinatorial treatment, reduces Mcl-1 protein level.

Whereas Mcl-1 regulates the PI-induced apoptosis, the PI3K/Akt signaling regulates both PIs- and TRAIL-induced apoptosis in MPM cells. Hyperactive PI3K/Akt signaling is commonly seen in MPMs due to frequent loss of PTEN expression [16] and is considered to be a negative prognosis marker for MPM [37]. This study reveals that the hyperactive Akt is associated with low sensitivity of MPM cells to PI-induced apoptosis. In addition, the sustained Akt activation, as seen in myr-Akt-transfected MPM cells, significantly reduces the sensitivity of tumor cells to PI alone, TRAIL alone, or even to the combinatorial treatment.

It has been well established that the Akt activity is most commonly regulated via phosphorylation and dephosphorylation. One consequence of the increased Akt phosphorylation/activation is the inhibition of its downstream protein GSK3β, which in turn can stabilize Mcl-1 protein from degradation by the proteasome. However, the present study did not suggest that Akt-GSK3β pathway is involved in regulating Mcl-1 stability in MPM cells. More likely, Akt and Mcl-1 are separately regulated in MPM cells and represent two independent resistance mechanisms to the PI-induced apoptosis, as demonstrated in the experiment using PI3K inhibitor LY294002 and Mcl-1 silencing in PI-treated NCI-H28 cells. It is possible that, during the regulation of the PI-induced apoptosis, the elevated Mcl-1 protein level blocks pro-apoptotic Bak protein [38], whereas the active Akt phosphorylate/sequester pro-apoptotic Bad protein from interacting with Bcl-X_L_[39].

However, the Akt protein and the Mcl-1 protein share the same feature of the PFM-directed protein cleavage in MPM cells following the combinatorial treatment. As seen in this study, the endogenous Akt protein was reduced through the caspase-dependent protein cleavage rather than the reduction of protein phosphorylation or gene transcription. The involvement of caspase 9 in the reduction of P-Akt has been seen previously in UVA-induced apoptosis [40], suggesting that cleavage-dependent mechanism may be an alternative mechanism to the phosphorylation mechanism, especially when the apoptotic machinery is fully activated by the PFM. Since several Akt isoforms exist in MPM cells including at least Akt1 and Akt3 and the antibodies used in the present study could not distinguish different isoforms [41], it is of great interest to determine which isoform is more involved in caspase-dependent regulation.

Consequently, following the PFM model, the increased caspase activities with the decreased resistance mechanisms of both Akt and Mcl-1 together ensure quick ultimate apoptotic cell death. More importantly, such robust proapoptotic activity exhibits a relative selectivity in MPM cells than in non-tumorigenic mesothelial cells. However, definitely, the underlying mechanism for the selectivity warrants further investigation.

## Conclusion

The present study demonstrates that the combinatorial treatment with TRAIL and PI induces a robust apoptosis more selectively in MPM cells than in normal mesothelial cell, therefore representing an effective candidate treatment for MPMs. The Akt protein serves as a new cleavage target of the PFM-directed caspase activation. In future clinical settings, the protein cleavage for both Akt and Mcl-1 may be used as effective efficacy marker to monitor the responses of MPM patients to the combinatorial treatment.

## Competing interests

The authors declare that they have no competing interests.

## Authors’ contributions

BZY, JAC and SHR have made substantial contributions to the conception and design of the study, the acquisition, analysis and interpretation of data. BZY has been responsible for critically drafting and revising the manuscript for all critical intellectual content. BZY has supervised the entire study and given the final approval of the revision to be published. MD, BHJ, JZW, and HR have made substantial contributions to the data acquisition and interpretation. All authors read and approved the final manuscript.

## Pre-publication history

The pre-publication history for this paper can be accessed here:

http://www.biomedcentral.com/1471-2407/13/140/prepub
